# Older adults’ home- and community-based care service use and residential transitions: a longitudinal study

**DOI:** 10.1186/1471-2318-12-44

**Published:** 2012-08-10

**Authors:** Ya-Mei Chen, Bobbie Berkowitz

**Affiliations:** 1 National Taiwan University, College of Public Health, Institute of Health Policy and Management, Room 633, No.1 7, Xu-Zhou Road, Taipei 100, Taiwan; 2 Columbia University, School of Nursing, 630 West 168th St., MC6, New York, NY 10032, USA

**Keywords:** Older adults, Home- and community-based care services (HCBS), Service use patterns, Policy implications, Residential transitions.

## Abstract

**Background:**

As Home-and Community-Based Services (HCBS), such as skilled nursing services or personal care services, have become increasingly available, it has become clear that older adults transit through different residential statuses over time. Older adults may transit through different residential statuses as the various services meet their needs. The purpose of this exploratory study was to better understand the interplay between community-dwelling older adults’ use of home- and community-based services and their residential transitions.

**Methods:**

The study compared HCBS service-use patterns and residential transitions of 3,085 older adults from the Second Longitudinal Study of Aging. Based on older adults’ residential status at the three follow-up interviews, four residential transitions were tracked: (1) Community-Community-Community (CCC: Resided in community during the entire study period); (2) Community-Institution-Community (CIC: Resided in community at T1, had lived in an institution at some time between T1 and T2, then had returned to community by T3); (3) Community-Community-Institution (CCI: Resided in community between at T1, and betweenT1 and T2, including at T2, but had used institutional services between T2 and T3); (4) Community-Institution-Institution (CII: Resided in community at T1 but in an institution at some time between T1 and T2, and at some time between T2 and T3.).

**Results:**

Older adults’ use of nondiscretionary and discretionary services differed significantly among the four groups, and the patterns of HCBS use among these groups were also different. Older adults’ use of nondiscretionary services, such as skilled nursing care, may help them to return to communities from institutions. Personal care services (PCS) and senior center services may be the key to either support elders to stay in communities longer or help elders to return to their communities from institutions. Different combinations of PCS with other services, such as senior center services or meal services, were associated with different directions in residential transition, such as CIC and CII respectively.

**Conclusions:**

Older adults’ differing HCBS use patterns may be the key to explaining older adults’ transitions. Attention to older adults’ HCBS use patterns is recommended for future practice. However, this was an exploratory study and the analyses cannot establish causal relationships.

## Background

Development of home- and community-based long-term care services (HCBS) in the United States began in the 1970s and expanded in the 1980s [1]. The passage of Medicaid home- and community-based service waivers in 1981 (Omnibus Budget Reconciliation Act of 1981, Medicaid 1915c) further fostered the development of HCBS [2]. The policy questions asked in past research studies were whether older adults’ use of HCBS reduced their use of institutional services such as nursing homes. However, as HCBS have become increasingly available, it has become clear that older adults transit through different residential statuses over time. Different transition patterns have been noted; older adults may move from communities to institutions or from institutions back to communities as various services meet their needs. The current question that should be asked is how older adults’ uses of HCBS interacts with their residential transitions, rather than whether HCBS reduces future nursing home admission. Some HCBS may be associated with older adults’ returning to communities or delaying use of institutional services, and these patterns are of interest to policy makers, people developing community services, and the wider society. As more older adults in need express their preferences for HCBS care, understanding how older adults’ HCBS use relates to their residential transitions would provide useful information about cost-effective ways to develop HCBS for particular populations [3].

HCBS in the United States are designed to offer older adults support that will allow them to age in their own communities [4]. The number of older adults in the United States is expected to double or triple when the baby boomers join the aging population [5,6]. Baby boomers have expressed a preference for staying at home or in communities [7-9]. They expect to receive care in their communities rather than via the intensive professional nursing care typical of public and private facilities. The need for HCBS will therefore increase greatly.

Researchers and service providers have found the association between older adults’ residential transitions and use of HCBS to be unclear. Many studies have investigated the effects of HCBS [2]. Findings were inconsistent. Some studies found that older persons who received HCBS entered nursing homes at a higher rate [10-12]; other studies found the opposite [13-15]. Some well-developed community-based service programs demonstrated to reduce nursing home use, but other programs showed HCBS to reduce institutionalization only in some groups [10,13]. Study findings suggest that HCBS is efficient when appropriately targeted [10,16,17]. Thus, the differing extents to which services are targeted is one explanation for the inconsistent findings.

Another possible explanation for the inconsistent findings in the literature is that residential transitions were ignored in past research studies. As HCBS have become increasingly available, older adults have been shown to transit through different residential statuses over time, such as from communities to institutions or from institutions back to communities. Therefore, in addition to examining the impact of targeting specific HCBS use on nursing home use in subgroups of older persons, research studies in this field should also take older persons’ residential transitions into consideration. Most previous research studies have failed to acknowledge this societal change. Understanding the interplay between older adults’ use of HCBS and their residential transition patterns could be key to developing an effective community-based long-term care system.

### Study purpose and research questions

The purpose of this study was to better understand the interplay between older adults’ HCBS use and their residential transitions. The study findings may provide insight for future development of community-based service systems that are more targeted and efficient. This study contributes to the literature by examining the relationships between HCBS use patterns and older adults’ residential transitions through data analysis of a longitudinal data set (The Second Longitudinal Study of Aging, LSOA II). This was the first study to address the relationship between older adults’ HCBS use and their residential transitions. It was an exploratory study, and did not intend to disentangle the causal relationships among HCBS use, disability status, and residential transitions. This study addressed these two related questions: (1) whether there are differences in service use among groups of older adults defined by residential transition patterns; and (2) which HCBS are associated with which residential transition patterns.

## Methods

This article explores the relationship between older adults’ HCBS use and their future residential transitions by analyzing a nationally representative dataset (The Second Longitudinal Study of Aging, 2002). The University of Washington Human Subjects Division approved this study.

### Data source- The second longitudinal study of aging (LSOA II)

The Second Longitudinal Study of Aging (LSOA II, 2002) was a collaborative effort of the National Center for Health Statistics (NCHS) and the National Institute on Aging (NIA). Using the LSOA II data, this study analyzed nationally representative civilian non-institutionalized persons aged 70 years or older. The LSOA II followed a stratified, multistage probability design that permitted continuous sampling of the target population. After baseline face-to face interviews in 1994 (Time 1 [T1]; N = 9,447), two follow-up interviews occurred using Computer Assisted Telephone Interviews [18]: one interview between 1997 and 1998 (Time 2 [T2]; N = 7,060), and one interview between 1999 and 2000 (Time 3 [T3]; N = 5,294). The overall response rate was over 74% [19]. Loss of respondents was due to attrition from death, hospitalization, and/or loss during tracking. The current study used one sample weight along with two sampling-related parameters (strata and psu) to account for the LSOA II’s sampling survey design.

### Study sample

A total of 3,085 older adults were included in the data analysis for this study. They were older adults who (1) had completed all three LSOA II interviews, and (2) had functional limitations at the baseline interview (T1), meaning that each had limitations in at least one disability in either Activity of Daily Living (ADLs) [20], Instrumental Activity of Daily Living (IADLs) [21], or Nagi’s functional limitation [22]. We selected the respondents with functional limitations because these older adults would be more likely than those who did not have any functional limitations to seek support services such as HCBS.

### Measures

The current study investigated associations between 13 different types of HCBS and four types of residential transition patterns. We further included 14 covariates, which were factors based on Anderson’s Health Behavioral Model (HBM).

### Types of HCBS

In general, each specific service within the category of HCBS can be assigned to one of two categories: nondiscretionary or discretionary. Nondiscretionary services typically require prescriptions from health care professionals. Physical therapy and skilled nursing care are examples of nondiscretionary services. In contrast, discretionary services are typically used as a matter of individual choice; homemaker/companion services and personal care services (PCS) are examples of nondiscretionary services [2,23,24].

The LSOA II data documented 13 services used by respondents between the T1 and T2 interviews. These 13 services were (a) senior centers, (b) Meals On Wheels, (c) meals at senior centers/facilities, (d) homemaker/companion services, (e) personal care services (PCS), (f) skilled nursing care, (g) physical therapy, (h) occupational therapy, (i) speech therapy, (j) dialysis, (k) tube feeding, (l) oxygen or respiratory therapy, and (m) hospice care. For the purpose of this study, the first five services were considered discretionary services; all the other services were considered nondiscretionary services. Two services (senior centers and meals at senior centers/facilities) were received outside the home, while all other services were received in the home. Because the T2 interview gathered the most detailed information on HCBS use, the current study used data from the T2 interview for information regarding HCBS use.

The LSOA II asked both 12-month and 2-year retrospective questions about HCBS use. The question asked at T2 interview for the first three services (services a through c) covered 12 months: “In the past 12 months, did you go to/use … [one of these services]?” [25]. For the remaining 10 services [services d through m], the questions covered 2 years. There were two questions: “Since [month/year of last interview] did you receive any health care services IN YOUR HOME? This would include skilled nursing care, physical or occupational therapy, assistance with medications or personal care needs, and any other services provided IN YOUR HOME by a visiting nurse, nursing assistant, home health aide, personal assistant, therapist, or homemaker”; and “Which of the following services did you receive? Did you receive (01) Skilled nursing care (02) Physical therapy (03) Occupational therapy (04) Speech therapy (05) Dialysis (06) Tube feeding (07) Personal assistant services (08) Homemaker/companion services (09) Oxygen / respiratory therapy (10) Hospice care.” [25]. All 13 HCBS variables were used to assess older adults’ use of HCBS between T1 and T2.

### Patterns of residential transition

**Figure 1:**
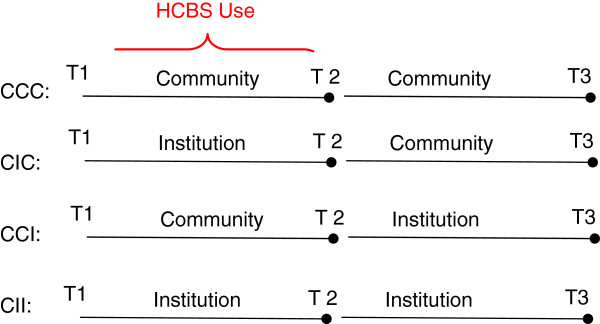
Older Adults’ HCBS Use and Residential Transitions From Time 1 to Time.

At each interview (T1, T2, and T3), each LSOA II respondent was living either in a home- and community-based setting (C) or in an institution (I). Home- and community-based settings included (a) single-family homes, (b) regular apartments, (c) retirement homes, (d) assisted living facilities, (e) supervised apartments, (f) group homes, (g) halfway houses, (h) boarding homes, and (i) developmental centers. Institutions included (j) nursing homes and (k) convalescent homes. All the older adults included in the LSOA II lived in communities (C) at the T1 interview. The questions asked at T2 and T3 interviews regarding residential status were these: “Is the place where you live a … [one of the 11 options described above]?” and “Since the last interview, have you been a resident in a nursing home/convalescent home?” (The Second Longitudinal Study of Aging—The Second Supplement on Aging, 1994). Each respondent who indicated residence in a nursing home, in answers to either of these two questions, was considered to have transited to an institution (I) during that period of time. Respondents whose answers did not indicate nursing home use were considered to be living in community (C). Respondents transitions between living arrangements were noted. The LSOA II collected data three times. Using these three time points, the current study defined four types of residential transitions: (1) CCC: older adults who resided in community from T1 to T3 and did not use any nursing home service during the entire study period (from 1994 to 2000); (2) CIC: older adults who resided in community at T1, had lived in an institution at some time between T1 and T2, including at T2, then had returned to community by T3 and had not used any nursing home services between T2 and T3; (3) CCI: older adults who resided in community between T1 and T2, including at T2, and did not use any nursing home services during this period of time, but had used nursing home services between T2 and T3, including at T3; and (4) CII: older adults who resided in community at T1 but in an institution at some time between T1 and T2, including at T2, and at some time between T2 and T3, including at T3. Figure 1 shows how the four groups of older adults were defined by type of residence from T1 interview to T3 interview, and when the HCBS use data was collected.

### Covariates from the health behavioral model

This study included 14 covariates based on factors from Anderson’s Health Behavioral Model (HBM), one of the most widely used behavioral models. The HBM posits that use of health services and residential transitions both are functions of three types of factors: factors that predispose people to use services, such as age, gender, marital status, race, region, and years of education; factors that enable or impede people’s use of such services, such as insurance information, family income, and size of family; and factors that affect people’s personal need for care, such as self-rated health, number of difficulties with Nagi’s Functional limitations, ADLs, and IADLs [20,26][27]. The HBM covariates, as well as older adults’ use of HCBS, were assessed as influences on older adults’ residential transition patterns [28-32]. Akaigbo and Wolinsky (2006) reported that older adults with a history of hospital use were associated with subsequent nursing home placement. As a result, we included among the covariates the variables of older adults being a hospital patient overnight either between T1 and T2 or between T2 and T3. All covariates were obtained from the T1 interview except for the variables of being a hospital patient overnight between T1 and T2 and being a hospital patient overnight between T2 and T3, which were drawn from the T2 and T3 interview data respectively.

### Missing values

Missing values in the study variables represented less than 5% of observations, with the exception of the HCBS variables (missing 8.8% to 15.6% of observations) and the income variable (missing 21% of observations). As a result, missing values from responses such as “not ascertained” and “don’t know or refused” were replaced using the Markov chain Monte Carlo method through the multiple-imputation procedure in LISREL 8.53 [33].

### Analysis

The first research question was whether there are differences in service use among groups of older adults defined by four residential transition patterns. Since each of the 13 HCBS was a dichotomous variable, we used logistic regression analysis, with predisposing, enabling, and need factors (Including age, gender, education level, family income, size of family, number of functional limitation, number of ADLs, Number of IADLs, and being in a hospital patient over night between T1 an T2 interviews and T2 and T3 interviews) controlled, to answer this question. As a result, a series of 13 logistic regression analyses was performed. Each analysis assessed the relationship between one HCBS and four residential transition pattern. Variables were considered significant at *P* value ≤ 0.05, 2-sided (SPSS manual, version 16).

The second research question was which HCBS are associated with which residential transition patterns. This study intended to explore (1) what services might help older adults to stay in their communities longer, (2) what services might help older adults move back to their communities once they have been institutionalized, and (3) whether using different services in combination might associate with different residential transition outcomes. To address this question, we used estimated marginal means produced by ANCOVA, controlled for age, gender, education level, family income, size of family, number of functional limitations, number of ADLs, number of IADLs, and overnight hospital stay(s) between T1 and T2 interviews and T2 and T3 interviews, and then created bar graphs depicting patterns of service use in different transition groups. To address the complex sample design used in LSOA II, the STATA 9.0 survey suite was used for statistical analysis.

## Results

Respondents’ ages ranged from 70 to 97 years, with a mean of 76.33. More than two thirds of the respondents were female (69.6%). The average number of years of education for all respondents was 11.16. Respondents’ family income ranged from $1,000 a year to more than $50,000 a year, with an average range from $15,000 to $16,999 a year. The majority of these older adults were living alone or with one family member (86.6%); the average number of people living in a respondent’s household was 1.82.

The older adults with functional limitations included in this analysis reported an average number of 1.06 (*SD* = 1.72) IADL disabilities, 0.95 (*SD* = 1.60) ADL disabilities, and 3.37 (*SD* = 2.40) functional limitations. About 46% of the sample received either discretionary or nondiscretionary services between T1 an dT2 interviews. The average number of services received was 0.34 (*SD* = 0.77) nondiscretionary services and 0.55 (*SD* = 0.83) discretionary services. The percentage of the sample in each transition group (defined by residential transition pattern) was as follows: CCC (N = 2589, 83.9%), CIC (N = 69, 2.2%), CCI (N = 283, 9.2%), and CII (N = 144, 4.2%). The following sections address the research questions’ findings. Table 1 and Table 2 provide detailed descriptive information about the 11 covariates and the 13 HCBS use in the four transition groups.

**Table 1:** Definition and distribution of health behavioural model (HBM) covariates

**HBM variables**	**Operational definitions**	**Mean (SD, Range)**	**Residential transition groups (N, Mean/Percentage)**			
**LSOAII N=3085**			**CCC N=2589**	**CIC N=69**	**CCI N=283**	**CII N=144**
**Predisposing Factors**						
Age	Years of age	76.40 (5.60, 69–97)	75.86 (N=2589)	77.88 (N=69)	78.9 (N=283)	80.48 (N=144)
Education	Years of education	11.04 (3.48, 0–18)	11.11 (N=2550)	11.35 (N=68)	10.77 (N=280)	10.17 (N=140)
Gender	Female	N = 2164 (70.1%)	N = 1785 (68.9%)	N = 52 (75.4%)	N = 209 (73.9%)	N = 118 (81.9%)
	Male	N = 921 (29.9%)	N = 804 (31.1%)	N = 17 (24.6%)	N = 74 (26.1%)	N = 26 (18.1%)
Racial	White	N = 2670 (87.1%)	N = 2226 (86.0%)	N = 63 (91.3%)	N = 251 (88.7%)	N = 130 (90.3%)
	Black	N = 328 (10.7%)	N = 284 (11.0%)	N = 6 (8.7%)	N = 26 (9.2%)	N = 12 (8.3%)
	Indian (American)	N = 13 (0.4%)	N = 12 (0.5%)		N = 1 (0.4%)	
	Chinese	N = 9 (0.3%)	N = 7 (0.3%)		N = 2 (0.7%)	
	Filipino	N = 10 (0.3%)	N = 9 (0.3%)		N = 1 (0.4%)	
	Japanese	N = 6 (0.2%)	N = 6 (0.2%)			
	Asian Indian	N = 3 (0.1%)	N = 3 (0.1%)			
	Guamanian	N = 1 (0.03%)	N = 1 (0.0%)			
	Other Race	N = 19 (0.6%)	N = 17 (0.7%)		N = 1 (0.4%)	N = 1 (0.7%)
	Multiple Race	N = 5 (0.2%)	N = 5 (0.2%)			
Marital status	Married	N = 1478 (47.9%)	N = 1300 (50.20%)	N = 28 (40.50%)	N = 103 (36.40%)	N = 47 (32.70%)
Self-rated health	Poor	N =284 (9.2%)	N = 228 (8.8%)	N = 9 (13.0%)	N = 25 (8.8%)	N = 22 (15.3%)
	Fair	N =726 (23.6%)	N = 600 (23.2%)	N = 16 (23.2%)	N = 71 (23.2%)	N = 39 (23.2%)
	Good	N =1131 (36.8%)	N = 948 (36.6%)	N = 30 (43.5%)	N = 104 (36.7%)	N = 49 (34.0%)
	Excellent	N =294 (9.6%)	N = 256 (9.9%)	N = 2 (2.9%)	N = 31 (11.0%)	N = 5 (3.5%)
**Enabling Factors**						
Household size	No. living in the same household	1.82 (0.96, 1–11)	1.85 (N=2589)	1.90 (N=69)	1.70 (N=283)	1.65 (N=144)
Family income	Higher scores indicate higher income (0 = less than $1,000; 26 = $50,000+)	16.11 (6.74, 0–26)	16.33 (N=2085)	16.94 (N=48)	14.93 (N=228)	13.85 (N=103)
**Need Factors**						
Nagi’s Functional limitations	No. of functional activities (e.g., climbing stairs, bending, lifting) unable to perform^a^ (0–10)	3.37 (2.40, 0–10)	3.22 (N=2589)	4.29 (N=69)	3.70 (N=283)	4.85 (N=144)
IADL disabilities	No. of IADLs unable to perform^c^ (0–8)	1.06 (1.72, 0–8)	0.92 (N=2587)	1.70 (N=69)	1.46 (N=283)	2.64 (N=144)
Hospital patient (T2)	Been a hospital patient overnight between T1 and T2	N=1832 (59.4%)	N = 1636 (63.2%)	N = 7 (10.1%)	N = 155 (54.8%)	N = 34 (23.6%)
Hospital patient (T3)	Been a hospital patient overnight between T2 and T3	N=1696 (55.0%)	N = 1567 (60.5%)	N = 33 (47.8%)	N = 47 (16.6%)	N = 49 (34.0%)
Difficulty with elders’ house (0-4)	N of Difficulty with elders’ house		1.41 (N=2589)	2.10 (N=69)	7.28 (N=283)	12.52 (N=144)
Unmet need in ADL (0-7)	N of ADLs needs more help		0.07 (N=2584)	0.03 (N=68)	0.18 (N=282)	0.34 (N=144)
Unmet need in IADL (0-8)	Number of IADL needs more help		0.13 (N=2584)	0.09 (N=68)	0.19 (N=282)	0.35 (N=144)

*Note.* All data from *The Second Longitudinal Study of Aging—The Second Supplement on Aging: 1994* (Version 2, No. 1, September 1998) [Data file]. Hyattsville, MD: National Center for Health Statistics. Available from http://www.cdc.gov/nchs/about/otheract/aging/lsoa2.htm.

^a^ From “An epidemiology of disability among adults in the United States,” by Nagi, 1976, *Milbank Memorial Fund Quarterly, 54 (4)*, pp. 439–476. ^b^From “Index of ADL ,” by Katz and Akpom, 1976,*Medical Care*, pp. 116–118. ^c^From “Assessment of older people; self-maintaining and instrumental activities of daily living,” by Lawton and Brody, 1969, *The Gerontologist,9*, pp. 179–186.

**Table 2:** Distribution of home- and community-based services (HCBS) variables

**HCBS Variables**	**Mean**	**SD**
GO TO SENIOR CENTER	0.23	0.40
MEALS ON WHEELS DELIVERED TO HOME	0.06	0.23
EAT AT SENIOR CENTER/FACILITY	0.13	0.32
RECEIVED SKILLED NURSING CARE	0.17	0.35
RECEIVED PHYSICAL THERAPY	0.10	0.29
RECEIVED OCCUPATIONAL THERAPY	0.04	0.17
RECEIVED SPEECH THERAPY	0.01	0.10
RECEIVED DIALYSIS	0.00	0.04
RECEIVED TUBE FEEDING	0.00	0.06
RECEIVED PERSONAL ASSISTANT SERVICES	0.09	0.27
RECEIVED HOMEMAKER/COMPANION SERVICES	0.06	0.22
RECEIVED OXYGEN / RESPIRATORY THERAPY	0.00	0.05
RECEIVED HOSPICE CARE	0.00	0

Note. All data from The Second Longitudinal Study of Aging—Wave 2 Survivor Data File (Version SF1.2, June 2002) [Data file]. Hyattsville, MD: National Center for Health Statistics. Available from http://www.cdc.gov/nchs/about/otheract/aging/lsoa2.htm.

Table 1 and Table 2 display definitions and descriptive statistics for included in the study.

### HCBS User by transition group

With all covariates controlled, older adults’ likelihood of receiving these services was found to differ significantly among the four groups: skilled nursing care (*p* < .001), physical therapy (*p* < .001), occupational therapy (*p* < .001), speech therapy (*p* < .001), Meals On Wheels (*p* < .001), eat at senior center/facility (*p* < .05), homemaker/companion services (*p* < .001), and PCS (*p* < .001), and meals at senior centers/facilities (*p* < .05) (Table 3).

**Table 3:** **Results of logistic regression analysis (*p*-value) and non-adjusted percentages regarding HCBS use**

	**HCBS use in each residential transition group**				***p*-value**
	**CCC (N = 2589)**	**CIC (N = 69)**	**CCI (N = 283)**	**CII (N = 144)**	
**DISCRETIONARY SERVICES**					
GO TO SENIOR CENTER	21.7% **(N = 561)**	20.3% **(N = 14)**	17.7% **(N = 50)**	3.5% **(N = 5)**	0.60
MEALS ON WHEELS DELIVERED TO HOME***	3.8% **(N = 99)**	11.6% **(N = 8)**	11.7% **(N = 33)**	27.1% **(N = 39)**	<0.001
EAT AT SENIOR CENTER/FACILITY*	11.3% **(N = 292)**	21.7% **(N = 15)**	12.0% **(N = 34)**	16.7% **(N = 24)**	0.027
RECEIVED PERSONAL CARE SERVICES***	5.7% **(N = 148)**	31.9% **(N = 22)**	12.7% **(N = 36)**	29.9% **(N = 43)**	<0.001
RECEIVED HOMEMAKER/COMPANION SERVICES***	3.6% **(N = 92)**	15.9% **(N = 11)**	11.0% **(N = 31)**	13.2% **(N = 19)**	<0.001
**NON-DISCRETIONARY SERVICES**					
RECEIVED SKILLED NURSING CARE***	12.0% **(N = 310)**	52.2% **(N = 36)**	18.0% **(N = 51)**	37.5% **(N = 54)**	<0.001
RECEIVED PHYSICAL THERAPY***	7.3% **(N = 189)**	33.3% **(N = 23)**	12.0% **(N = 34)**	24.3% **(N = 35)**	<0.001
RECEIVED OCCUPATIONAL THERAPY***	2.0% **(N = 51)**	17.4% **(N = 12)**	6.4% **(N = 18)**	11.1% **(N = 16)**	<0.001
RECEIVED SPEECH THERAPY***	0.7% **(N = 19)**	4.3% **(N = 3)**	1.1% **(N = 3)**	2.8% **(N = 4)**	<0.001
RECEIVED DIALYSIS	0.2% **(N = 5)**	0.0% **(N = 0)**	0.4% **(N = 1)**	0.0% **(N = 0)**	0.768
RECEIVED TUBE FEEDING	0.3% **(N = 9)**	2.9% **(N = 2)**	0.0% **(N = 0)**	1.4% **(N = 2)**	0.19
RECEIVED OXYGEN / RESPIRATORY THERAPY	0.2% **(N = 5)**	1.4% **(N = 1)**	0.4% **(N = 1)**	0.7% **(N = 1)**	0.386

*p < 0.05. **p < 0.01. ***p < 0.001.

Note 1. All data from The Second Longitudinal Study of Aging—The Second Supplement on Aging: 1994 (Version 2, No. 1, September 1998); Wave 2 Survivor Data File (Version SF1.2, June 2002); Wave 3 Survivor Data File (Version SF2.1, October 2002) [Data file]. Hyattsville, MD: National Center for Health Statistics. Available from http://www.cdc.gov/nchs/about/otheract/aging/lsoa2.htm.

Note 2. *C* = Community: Home- and community-based settings are settings either in a housing unit or in a facility which provides residents with autonomy and control over their living and service arrangements. I = Institution: Residential settings in units that are neither self-contained nor self-sufficient are considered institutions and units in such settings are often shared by nonrelated residents (including settings like nursing homes and convalescent or rest homes).

### HCBS Use patterns and residential transitions

**Figure 2:**
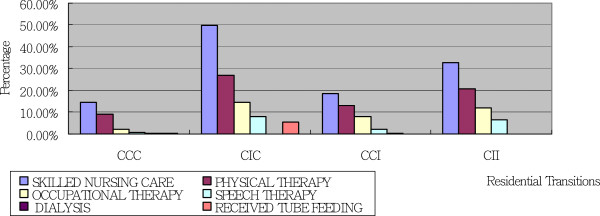
**Patterns of Nondiscretionary Services Use by Residential Transitions Using Marginal Means Produced by Analysis of Covariance.** Note. C = Community; I = Institution. Age, Gender, Education level, Family income, Size of family, Number of Functional Activity, Number of ADLs, Number of IADLs, and Being a Hospital Patient Overnight between T1 and T2 interviews and T2 and T3 interviews were adjusted using Analysis of Covariance.

**Figure 3:**
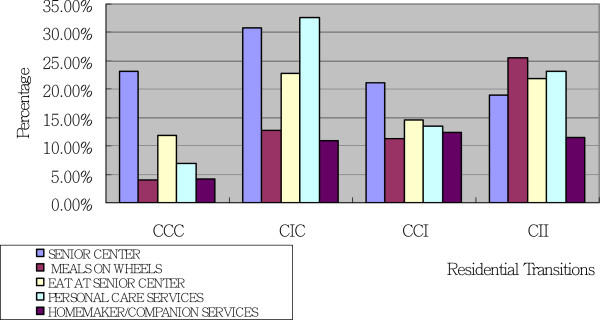
**Patterns of Discretionary Services Use by Residential Transitions Using Marginal Means Produced by Analysis of Covariance.** Note. C = Community; I = Institution. Age, Gender, Education level, Family income, Size of family, Number of Functional Activity, Number of ADLs, Number of IADLs, and Being a Hospital Patient Overnight between T1 and T2 interviews and T2 and T3 interviews were adjusted using Analysis of Covariance.

HCBS use patterns indicate how using services or services in combination might relate to different residential transitions. The groups of older adults included in this study demonstrated different service use patterns. The older adults in all four groups demonstrated similar patterns of nondiscretionary services use, yet different numbers of older adults in each group used these services. In contrast, each of the four different groups demonstrated a different pattern of discretionary services use. Figures 2 and 3 present older adults’ use of nondiscretionary and discretionary services respectively.

Use patterns for each individual nondiscretionary service were similar for older adults in all four groups. The nondiscretionary service most commonly received was skilled nursing care, followed by physical therapy and occupational therapy. Among the adults in the CIC group, a greater percentage than in the other groups used each nondiscretionary service. The services most commonly received at home by the older adults in the CIC group were skilled nursing care (49.9%), physical therapy (27.0%), and occupational therapy (14.5%). These findings require further discussion (Figure 2).

As for discretionary services use, the use patterns for each individual discretionary services were different for older adults in all four groups. Older adults in the CCC group seemed to use senior centers (23.2%) more commonly than other discretionary services (4.0%-11.7%). The older adults in the CIC group most commonly used senior centers (30.8%) and PCS (32.7%). It was also common for older adults in the CIC group to have meals at senior centers (22.9%).

The pattern of discretionary services use for older adults in the CCC and CCI groups was similar. Older adults in both groups commonly used senior centers (CCC: 23.2%; CCI: 21.1%). However, when compared to the CCC group, more older adults in the CCI group used other four services, such as Meals On Wheels or PCS. Older adults in the CCI group used discretionary services, except senior center services, almost equally (11.2%–14.5%).

Older adults in the CII group most commonly used the Meals On Wheels service (25.5%), which was the service least commonly used by the older adults in the other three groups. Older adults in the CII group also commonly used another meal service, meals at senior centers (21.9%), as well as many other discretionary services, such as PCS (23.1%). Unlike the older adults in the other three groups, who seemed to use one or two particular services most often, the older adults in the CII group used many kinds of discretionary services. These service use patterns will be discussed further below.

## Discussion

This study’s findings provide some explanations for the inconsistent research findings in the literature regarding the association between older adults’ HCBS use and their future residential transitions. In the literature, HCBS have been examined either as a unified category or as individual services. Previous research studies have not examined how outcomes from one service might be supported by use of other services, or how using different combinations of services might result in different outcomes regarding residential transitions. Our findings show the possibility that older adults’ use of different HCBS, and different patterns of HCBS use, might interact with older adults’ residential transitions. These findings have some value for future research and policy making for older adults living in communities.

### Nondiscretionary services and residential transitions

Among the nondiscretionary services, there were significant differences among the four groups in the use of skilled nursing care, physical therapy, and occupational therapy. Figure 2 shows that the older adults in the CIC group were the most likely to use these three services. It is likely that the older adults in this group were introduced to these services in an early stage of disability and therefore knew how to access the services again once institutionalization was no longer required. Knowledge of service accessibility may have supported these adults in returning to their communities [34]. Our study findings provide some empirical support to Bradley and colleagues’ expanded Health Behavioral Model, in that previous experiences of using long-term care services could increase older adults’ knowledge and perceived control of accessing the services when they next need them [34]. Such knowledge and attitude could empower older adults to return to communities from institutions.

On the other hand, we cannot ignore the possibility that older adults might have these services ordered for them upon leaving an institution. The literature suggests that older adults who go to nursing homes for short-term rehabilitation after an acute event may use more occupational therapy or physical therapy after returning to their communities [35]. However, both scenarios point out that using nondiscretionary services, no matter whether before or after institutionalization, may help older adults to return to communities. Fewer older adults in the CII group used these nondiscretionary services between T1 and T2, compared to the CIC group, and these older adults tended to use institutional services again between T2 and T3. Research studies found that formal community services, when appropriately targeted to certain subgroups of older adults with functional limitations, appear to be significantly associated with reduced risk of nursing home use [10,36]. The findings from this study provide further insights: (a) skilled nursing care, physical therapy, occupational therapy, and speech therapy may be helpful in helping older adults to return to communities from institutions, and thus may be more closely related to reducing nursing home use; and (b) there may be ideal times to provide services to older adults in order to achieve these results. For example, early introduction to occupational services may enhance older adults’ ability to return to community after institutionalization, as in the CIC group. This requires further investigation.

### Discretionary services and residential transitions

The four groups of older adults used significantly different types of discretionary services. Different key services, and services that support these key services, were identified for each of the four groups. Older adults in the CCC, CIC, and CCI groups commonly used senior centers. Older adults in the CIC group commonly used both senior centers and PCS. Older adults in the CII group commonly used meal services (both Meals On Wheels and meals at senior centers) as well as other discretionary services, such as PCS. We further discuss these service use patterns below.

Although the use of senior centers was not significantly different among the four transition groups, senior centers were commonly used by older adults in the CCC, CIC, and CCI groups. It is possible that use of senior centers, rather than directly facilitating the ability to stay in community, is instead a characteristic of older adults who are more outgoing and more willing to connect with others. These personal characteristics in themselves may eventually enable older adults to continue to stay in their communities (like those in the CCC group), to community after being institutionalized (like those in the CIC group), or to stay in community longer (like those in the CCI group). Personal characteristics can influence the type of care received [37], while using senior centers can moderate the effects of stress on distress and has been found to positively associate with older adults’ physical and psychological well-being [38-40]. It may be a combination of these effects that provides older adults with functional limitations who use senior centers the strength to stay healthy and to either continue to live in their communities or live in community longer. The mechanism that underlies these adults’ consequent ability to remain living in communities merits further investigation.

Older adults’ use of senior centers in combination with different other services may associate with different future residential transitions as well. Older adults who used senior centers but not other services (like the older adults in the CCC group) tended to continue to stay in communities. It is possible that this group used senior centers for recreational purposes only, which is good for maintaining psychological health, and they did not need to use other services [38-40]. It could be that when these adults develop needs for and begin to use other services (like the older adults in the CCI group), future nursing home admission becomes possible. Older adults in the CCI group used all other types of discretionary service equally and only slightly less commonly than senior centers. This could indicate that senior center services used along with many other HCBS, as seen in the CCI group, helps older adults manage to stay in communities longer when they developed needs.

If older adults learn to access PCS (like the older adults in the CIC group) early on, then use of senior centers in combination with PCS may enable them to return to communities after being institutionalized (like the older adults in the CIC group). PCS stands out as the most commonly used service by the older adults in the CIC group. Studies have found that providing PCS to older adults reduced their use of nursing facilities and supported them to remain in communities [35,41]. Findings in the current study provide a potential explanation regarding the mechanism by which using PCS supports older adults to remain in communities: that using PCS along with senior centers, skilled nursing care, physical therapy, occupational therapy, and speech therapy, like the CIC group did, might enable older adults to move back to communities from institutions.

In the CII group, PCS was also used by many older adults; however, the pattern of HCBS use in this group was quite different from that in the CIC and CCI groups. In the CII group, Meals On Wheels was the most common service used, followed by PCS and meals at senior centers/facilities. This pattern seemed to be associated with possible transition to an institution in the future. It could be that older adults in the CII group, whether due to physical limitations or lack of access to appropriate kitchen facilities, had difficulties preparing food for themselves, and that this characteristic is associated with future long-term or frequent use of institutional care services. Case managers or health care professionals who notice this pattern of HCBS use might consider recommending institutional care, instead of trying to keep these older adults in communities.

### Policy implications

What kind of services to provide and when has always been of great interest to policy makers [42-44]. The current study findings not only shed some light on HCBS service provision and policy development, but also point out the importance of rethinking the relationships between different HCBS and older adults’ residential transitions. With the use of HCBS, older adults may be able to continue staying in communities, move back to their communities from institutions, or stay in their communities longer before moving into an institution. The U.S. government has been supporting older adults living in communities, and also supporting those who live in nursing homes but could live in communities to transfer back to communities (Mor, 2007). Our study findings could provide insight for helping both the older population and the U.S. government to achieve their goals.

Although the mechanism for how different service use or service use in combinations interact with older adults’ residential transitions will require further study, our current findings provide some information for policy makers and case managers as well as reason to pay attention to older adults’ patterns of HCBS use. Our study findings also provide a potential explanation for the inconsistent findings in the literature regarding the effects of HCBS use: It is possible that using HCBS in different combinations may result in different outcomes. Past studies had proposed that different HCBS may have different effects on nursing home use for different subgroups of older adults [10]. Our study findings further pointed out that different combinations of HCBS use are related to different residential transitions. Further study to identify the characteristics of the older adults in these four different residential transition groups will be important for future policy making and practice.

### Research implications

Another contribution of the current study is the acknowledgment of older adults’ residential transitions from a longitudinal perspective while studying the effect of older adults’ HCBS use. A review of trends in the quantitative analysis of social science data on aging during the past half century shows that cross-sectional analysis remains the single most frequent type of study design [45], particularly in studies examining the effect of HCBS [17,31,32,46-48]. This type of study design does not allow researchers to study the dynamics of older adults’ residential transitions and use of HCBS and may have contributed to the inconsistent research findings in research studies that have examined the effect of HCBS. Thus, longitudinal study design is recommended for future research in aging services. In addition, our study findings recommend that future research in HCBS use among older adults include at least three time points, to study the transitions. As HCBS have become increasingly available, older adults have been shown to transit through different residential statuses over time. Most previous research studies have failed to acknowledge this societal change. Understanding the interplay between older adults’ use of HCBS and their residential transition patterns could be key to developing an effective community-based long-term care system. Examining the associations between older adults’ residential transitions and HCBS use from a longitudinal perspective could provide further insight into the inconsistent findings in the literature and policy and practice implications [10-15][10,16,17].

### Limitations

Several study limitations warrant discussion. First, the results reported here are subject to the limitations of variable availability in the data set. Service utilization was one of the most important outcome measures in this study. Receipt of HCBS is related to service availability and depends on the distribution system, not on the mobility of the user. A service that is not available cannot be used [49]. However, the variable of service availability was not recorded in the national data set used in this study and therefore could not be studied. Other service variables not included in the data set also could not be studied. For example, adult day care service, which was a common HCBS available in communities, was not surveyed at the T2 interview. Therefore, we were not able to study how this service might influence older adults’ residential transitions. We would also like to note that the four residential transition patterns included as variables in the current study do not capture all of the possible transitions between observation points, and thus in some sense these patterns are artificially determined by the design of the LSOA II.

We also questioned whether the older adults in the CIC group were those who were hospitalized for an acute condition and sent to a nursing home for a few weeks of recovery, with high probability of return to the community. We ran some analyses to compare the number of days in institutions and the frequency of institutional service use for the older adults in the CIC and CII groups. The CIC and CII groups had a similar ratio of total institutional days per institution admission during the period from T1 and T2 (1.08 ± 0.66 days per admission to an institution vs. 1.44 ± 1.78 days per admission), but very different ratios of days in institutions per admission during the period from T2 and T3 (0 days per admission to an institution vs. 16.66 ± 13.34 days per admission). It is intriguing to consider whether these two groups’ use of different HCBS (or different combinations of HCBS) between T1 and T2 could be associated with their differential use of institutional services at a later time. However, many factors may play a role in whether a group of older adults changes from short-term users to nonusers of institutional services (CIC), or from short-term to frequent/long-term users of institutional services. Studies to further investigate these factors are recommended.

We noted that individuals in the study sample tended to have more functional limitations and ADL and IADL disabilities than the overall LSOA II population. The functional status of the older adults in the study sample was similar to, but a little less disabled than, that of those LSOA II respondents who were not able to participate in the T2 and T3 surveys: our study sample had more Nagi’s functional limitations, which are considered light disabilities, and less ADL and IADL disabilities, which are considered more severe disabilities. Therefore, the current study’s findings may be generalizable only to older adults with a light to moderate level of functional disabilities. Further investigation is merited for service use patterns in severely disabled older populations as well as service use patterns among respondents who died or dropped out prior to completion of the LSOA II study, because they may have very different service use patterns than did the older adults included in this study.

## Conclusions

Research studies in the literature about the relationship between older adults’ HCBS and nursing home use were most likely to focus on the effect of single service types, such as skilled nursing care. The power of the combining different HCBS services has not been examined. The current study pointed out not only several key services but also that combination of key and other services are associated with different directions in residential transitions. These findings provide insight for future HCBS policy development and long-term care research. We suggest that further investigation is merited not only on the impact of specific types of community services on subsequent nursing home use, as recommended by Jette and colleagues [10], but also on the supportive effect of combining different HCBS to the key individual services. Further policy investment on developing key services identified in the current study is also recommended.

However, this was an exploratory study and the analyses cannot establish causal relationships. Future studies are needed to further investigate the causal relationships between older adults’ HCBS use and their residential transitions.

## Abbreviations

ADLs, Activity of daily living; IADLs, Instrumental activity of daily living; HCBS, Home-and community-based services; PCS, Personal care services; I, Living in institution; C, Living in community; CCC, Older adults who resided in community from T1 to T3 and did not use any nursing home service during the entire study period; CIC, Older adults who resided in community at T1 had lived in an institution at some time between T1 and T2, including at T2, then had returned to community by T3 and had not used any nursing home services between T2 and T3; CCI, Older adults who resided in community between T1 and T2 including at T2, and did not use any nursing home services during this period of time, but had used nursing home services between T2 and T3, including at T3; CII, Older adults who resided in community at T1 but in an institution at some time between T1 and T2 including at T2, and at some time between T2 and T3, including at T3; LSOA II, The second longitudinal study of aging; HBM, Health behavioral model.

## Competing interests

The authors had no conflicts of interest with respect to the authorship or the publication of this article.

## Authors’ contributions

Chen and Berkowitz formulated the idea and led the design, preparation of the manuscript, and revising manuscript critically for the core intellectual content. Chen conducted the statistical analysis and interpretation of the data. All authors read and approved the final manuscript.

### Funding

The study was supported by Hester McLaws’s Nursing Scholarship from the University of Washington School of Nursing. The findings are entirely the responsibility of the authors and do not reflect the views of the funding agencies.

## Pre-publication history

The pre-publication history for this paper can be accessed here:

http://www.biomedcentral.com/1471-2318/12/44/prepub

## References

[B1] GAO Long-Term Care: Current Issues and Future Directions 1995 United States General Accounting Office, Washington, D.C 1 33

[B2] Miller NA Ramsland S Goldstein E Harrington C Use of Medicaid 1915(c) home- and community-based care waivers to reconfigure state long-term care systems Med Care Res Rev 2001 58 1 100 119 11236230 10.1177/107755870105800106

[B3] Fischer LR Green CA Goodman MJ Brody KK Aickin M Wei F Phelps LW Leutz W Community-based care and risk of nursing home placement Medical care 2003 41 12 1407 1416 10.1097/01.MLR.0000100587.51573.7A 14668673

[B4] Kane RA Kane RL Ladd RC The Heart of Long-Term Care 1998 Oxford University Press, New York

[B5] GAO Long-Term Care: Baby Boom Generation Increases Challenge of Financing Needed Services 2001 United Stated General Accounting Office, Washington, D.C

[B6] Pryor D Durenberger D GAO HEHS-95-16: Long Term Care Population. Washington, D.C 1994 Health, EducatGeneral Accounting Office 1 24

[B7] Mollica R Coordinating services across the continuum of health, housing, and supportive services J Aging Health 2003 15 1 165 188 10.1177/0898264302239022 12611413

[B8] Eckert JK Morgan LA Swamy N Preferences for receipt of care among community-dwelling adults J Aging Soc Policy 2004 16 2 49 65 10.1300/J031v16n02_04 15148044

[B9] Langa KM Chernew ME Kabeto MU Katz SJ The explosion in paid home health care in the 1990s: Who received the additional services? Medical care 2001 39 2 147 157 10.1097/00005650-200102000-00005 11176552

[B10] Jette AM Tennstedt S Crawford S How does formal and informal community care affect nursing home use? The journals of gerontology Series B, Psychological sciences and social sciences 1995 50 1 S4 S12 10.1093/geronb/50b.1.s4 7757829

[B11] Hanley RJ Alecxih LM Wiener JM Kennell DL Predicting elderly nursing home admissions. Results from the 1982–1984 National Long-Term Care Survey Res Aging 1990 12 2 199 228 10.1177/0164027590122004 2359874

[B12] McFall S Miller BH Caregiver burden and nursing home admission of frail elderly persons J Gerontol 1992 47 2 S73 S79 1538078 10.1093/geronj/47.2.s73

[B13] Wooldridge J Schore J The evaluation of the National Long Term Care Demonstration. 7. The effect of channeling on the use of nursing homes, hospitals, and other medical services Health Serv Res 1988 23 1 119 127 3130323 PMC1065492

[B14] Eng C Pedulla J Eleazer GP McCann R Fox N Program of All-inclusive Care for the Elderly (PACE): an innovative model of integrated geriatric care and financing J Am Geriatr Soc 1997 45 2 223 232 9033525 10.1111/j.1532-5415.1997.tb04513.x

[B15] Mitchell G Salmon JR Polivka L Soberon-Ferrer H he relative benefits and cost of Medicaid home- and community-based services in Florida Gerontologist 2006 46 4 483 494 10.1093/geront/46.4.483 16921002

[B16] Chen YM A community-based long-term care model for the U.S. elderly. Unpublished doctoral dissertation 2004 University of Washington, Seattle

[B17] Greene VL Lovely ME Ondrich JI Do community-based long-term care services reduce nursing home use? A transition probability analysis J Hum Resour 1993 28 2 297 317 10.2307/146205

[B18] The Second Longitudinal Study of Aging The Second Supplement on Aging: 1994 (Version 2, No. 1, September 1998); Wave 2 Survivor Data File (Version SF1.2, June 2002); Wave 2 Decedent Data File (Version DF1.1, August 2002); Wave 3 Survivor Data File (Version SF2.1, October 2002); Wave 3 Decedent Data File (Version DF2.1, December 2002) 2002 [cited 2002; Data files] Available from: http://www.cdc.gov/nchs/about/otheract/aging/lsoa2.htm

[B19] The Second Longitudinal Study of Aging The Second Supplement on Aging: Wave 3 Survivor Questionnaire (Version SF2.1, October 2002) 2002 [cited 2002; Coding Book files] Available from: http://www.cdc.gov/nchs/about/otheract/aging/lsoa2.htm

[B20] Katz S Akpom CA Index of ADL Medical care 1976 14 5 Suppl 116 118 132585 10.1097/00005650-197605001-00018

[B21] Lawton MP Brody EM Assessment of older people; self-maintaining and instrumental activities of daily living Gerontologist 1969 9 179 186 10.1093/geront/9.3_Part_1.179 5349366

[B22] Nagi SZ An epidemiology of disability among adults in the United States Milbank Mem Fund Q Health Soc 1976 54 4 439 467 10.2307/3349677 137366

[B23] Miller NA Medicaid 2176 home and community-based care waivers: the first ten years Health Aff (Millwood) 1992 11 4 162 171 10.1377/hlthaff.11.4.162 1483635

[B24] Home and Community-Based Services 1915 (c) waivershttp://www.hcfa.gov/medicaid/hpg4.htm

[B25] The Second Supplement on Aging: Wave 3 Survivor Questionnaire (Version SF2.1, October 2002) http://www.cdc.gov/nchs/about/otheract/aging/lsoa2.htm

[B26] Katz S Downs TD Cash HR Grotz RC Progress in development of the index of ADL Gerontologist 1970 10 1 20 30 10.1093/geront/10.1_Part_1.20 5420677

[B27] Kane RA Kane RL Assessing the elderly: A practical guide to measurement 1981 Lexington Books, Lexington, MA

[B28] Andersen R Newman JF Societal and individual determinants of medical care utilization in the United States Milbank Mem Fund Q Health Soc 1973 51 1 95 124 10.2307/3349613 4198894

[B29] Andersen RM A Behavioral Model of Families' Use of Health Services. Chicago 1968 University of Chicago, Center for Health administration Studies

[B30] Andersen RM Revisiting the behavioral model and access to medical care: Does it matter? J Health Soc Behav 1995 36 1 1 10 10.2307/2137284 7738325

[B31] Krout JA Oggins J Holmes HH Patterns of service use in a continuing care retirement community Gerontologist 2000 40 6 698 705 10.1093/geront/40.6.698 11131086

[B32] Mitchell J Krout JA Discretion and service use among older adults: The behavioral model revisited Gerontologist 1998 38 2 159 168 10.1093/geront/38.2.159 9573660

[B33] Jöreskog KG Sörbom D LISREL8: Structural equation modeling with the SIMPLIS cammand language 1993 Lawrence Erlbaum Associates, Hillsdale, NJ

[B34] Bradley EH McGraw SA Curry L Buckser A King KL Kasl SV Andersen R Expanding the Andersen model: the role of psychosocial factors in long-term care use Health Serv Res 2002 37 5 1221 1242 10.1111/1475-6773.01053 12479494 PMC1464025

[B35] Dale SB Brown R Reducing nursing home use through consumer-directed personal care services Medical care 2006 44 8 760 767 10.1097/01.mlr.0000218849.32512.3f 16862038

[B36] Greene VL Lovely ME Ondrich JI The cost-effectiveness of community services in a frail elderly population Gerontologist 1993 33 2 177 189 10.1093/geront/33.2.177 8468010

[B37] Braun KL Rose CL Finch MD Patient characteristics and outcomes in institutional and community long-term care Gerontologist 1991 31 5 648 656 10.1093/geront/31.5.648 1778491

[B38] Farone DW Fitzpatrick TR Tran TV Use of senior centers as a moderator of stress related distress among Latino elders J Gerontol Soc Work 2005 46 1 65 83 10.1300/J083v46n01_05 16338885

[B39] Wheaton B Models for the stress-buffering functions of coping resources J Health Soc Behav 1985 26 4 352 364 10.2307/2136658 4086758

[B40] Aday RH Kehoe GC Farney LA Impact of senior center friendships on aging women who live alone J Women Aging 2006 18 1 57 73 10.1300/J074v18n01_05 16635950

[B41] Chen YM Thompson EA Understanding factors that influence success of HCBS in keeping older adults in community settings J Aging Health 2010 22 3 267 291 10.1177/0898264309356593 20103687

[B42] Allen KG Elderly Individuals Could Find Significant Variation in the Availability of Medicaid Home and Community Services. Washington, D.C 2002 GAO

[B43] GAO Long-Term Care: Federal Oversight of Growing Medicaid Home and Community-Based Waivers Should Be Strengthened. Washington D.C 2003 United States General Accounting Office

[B44] GAO LONG-TERM CARE: Availability of Medicaid Home and Community Services for Elderly Individuals Varies Considerably. Washington, D.C 2002 United States General Accounting Office

[B45] Ferraro KF Kelley-Moore JA A half century of longitudinal methods in social gerontology: Evidence of change in the journal The journals of gerontology Series B, Psychological sciences and social sciences 2003 58 5 S264 S270 10.1093/geronb/58.5.S264 14507936

[B46] Feder J Komisar HL Niefeld M Long-term care in the United States: An overview Health Aff (Project Hope) 2000 19 3 40 56 10.1377/hlthaff.19.3.40 10812780

[B47] Mitchell J Service awareness and use among older North Carolinians J Appl Gerontol 1995 14 193 209 10.1177/073346489501400204

[B48] Rabiner DJ Patterns and predictors of noninstitutional health care utilization by older adults in rural and urban America J Rural Health 1995 11 4 259 273 10.1111/j.1748-0361.1995.tb00424.x 10153686

[B49] Logan JR Spitze G Informal support and the use of formal services by older Americans J Gerontol 1994 49 1 S25 S34 8282986 10.1093/geronj/49.1.s25

